# Quasi‐independent monitor unit calculation for intensity modulated sequential tomotherapy

**DOI:** 10.1120/jacmp.v3i2.2577

**Published:** 2002-03-01

**Authors:** Jen‐San Tsai, Mark J. Engler, James Liu

**Affiliations:** ^1^ Department of Radiation Oncology Tufts University School of Medicine Boston, 136 Harrison Avenue Boston Massachusetts 02111; ^2^ New England Medical Center Hospitals (a Lifespan Partner) Boston Massachusetts 02111

**Keywords:** independent calculation, monitor unit, sequential tomotherapy, intensity modulated radiation therapy

## Abstract

The number of linac monitor units (MU) from intensity modulated sequential tomotherapy (IMST) is substantially larger than the MU delivered in conventional radiation therapy, and the relation between MU and dose is obscure due to complicated variation of the beam intensities. The purpose of this work was to develop a practical method of verifying the MU and dose from IMST so that the MU of each arced beam could be double‐checked for accuracy. MU calculations for 41 arced beams from 14 IMST patients were performed using the variables of vane open fraction time, field size, target depth output factor, TMR, and derived intensity distribution. Discrepancy between planned and checked MU was quantified as $100({\text{MU}}_{\text{cal}}-{\text{MU}}_{\text{plan}})/{\text{MU}}_{\text{plan}}$ percent. All 41 discrepancies were clustered between −5% to +5%, illustrated in a Gaussian‐shaped histogram centered at −1.0±3.5% standard deviation indicating the present MU calculations are in agreement with the planned expectations. To confirm the correctness of the present calculated MUs of the IMST plans, eight of the calculated IMST plans are performed dose verifications using their hybrid plans, which are created by transporting patient's IMST plan beams onto a spherical polystyrene Phantom for dose distribution within the Phantom. The dose was measured with a 0.07 cc ionization chamber inserted in the spherical Phantom during the hybrid plan irradiation. Average discrepancy between planned and measured doses was found to be 0.6±3.4% with single standard deviation uncertainty. The spread of the discrepancies of present calculated MUs relative to their planned ones are attributed to uncertainties of effective field size, effective planned dose corresponding to each arc, and inaccuracy of quantification of scattered dose from adjacent arced beams. Overall, the present calculation of MUs is consistent with what derived from treatment plans. Since the MUs are verified by actual dose measurements, therefore the present MU calculation technique is considered adequate for double‐checking planned IMST MUs.

PACS number(s): 87.53.–j, 87.66.–a

## I. INTRODUCTION

Intensity modulated sequential tomotherapy (IMST, NOMOS Corp., Sewickley, PA) is the technique using both field shaping and intensity variation to configure a dose distribution 3D conformal to the target to minimize the dose toxicity to the normal tissues adjacent to the target. Unfortunately, at the present time, there is no technology that enables the radiation source itself to achieve a well‐controlled beam intensity pattern in both time sequence and specified space. Therefore, current beam intensity variation mainly relies on an external apparatus to manipulate the opening of the beam either partially or fully in time space. Since the radiation beam maintains its constant monitor units (MU) delivery rate, this implies that some radiation beams, which are presumably to irradiate some specific anatomic site and the neighboring tissues, are sometimes blocked off or partially attenuated. Thus to deliver a prescribed dose to the patient, the IMST linac runs a substantially larger MU and simultaneously far more unpredictably than the MU delivered in conventional radiation therapy. In fact, the relationship between MU and dose for IMST is determined by a set of multiple intertwined procedures ranging from imaging, computer networking, treatment planning, optimization, and treatment delivery^1^ ^–^ ^9^ and is entirely obscured to the radiation physicist, radiation oncologist, and therapist delivering the treatment. In compliance with the QA of dose delivery, especially in IMST dose escalation treatments, AAPM report #38,^10^ accompanied by the policy of Radiological Physics Center, invokes the importance and necessity of double checking of MUs prior to treatment in radiotherapy. In the past, Tsai *et al*.,^11^ Low *et al*.,^12^ Teh *et al*.,^13^ and Verellen *et al*.,^14^ have performed integrally IMST plan verification, indirectly fulfilling some MUs second check, using a humanoid phantom prior to IMST treatment. This integrated plan verification procedure requires about four hours per patient. To reduce the time required for IMST plan verification, some QA procedure may not have to proceed on every individual patient because of known slowly varying quality condition of the radiotherapy mode without pose any significant risk in a short period of time. However, to maintain the high vigilant QA, MUs second check, being directly related to dose delivery and varying from patient to patient, is perhaps the non‐compromising alternative QA needed. This article addresses this issue.

Reports of MU calculations for verifications of multi‐leaf collimator (MLC) intensity‐modulated radiation therapy (IMRT) as a dosimetry QA have been done by Boyer *et al*.^15^ and Kung *et al*.^16^ Recently, Ayyangar *et al*.^17^ have done an independent dose calculation for MIMiC collimated IMRT (NOMOS Corp., Sewickley, PA) by using the plan optimized intensity beamlets and subsequently repeated dose calculation on the same anatomy by simple dosimetric formulas to check the plan dose distribution. This article, in the same wavelength, independently develops a MU calculation based on some modeled parameters is to dedicate for IMST using the MIMiC collimator. In the present article, the MU calculation is based on the input data composed of measured values and some modeled estimations, for example the average dose, which is inevitably embedded in some intrinsic uncertainty. This implies that we are dealing with the macroscopic, instead of voxel‐wised microscopic quantities. Since the purpose of the present work is for vigilant checking of the MUs derived from the IMST plan, our task is to scrutinize the MUs of IMST plan so that they are not far off from the present calculated ones. In other words, a high accuracy of the calculated MU may not be essential albeit we are further exploring for better accuracy.

## II. MATERIALS AND METHODS

### A. Glossary of terms

To help readers understand the article, some terminology, acronyms, and symbols involved with the article are specified, including their units as follows:

**Table:** 

Coordinates system	The coordinate system (*x,y,z*) of NOMOS' IMST and couch index are sketched in Fig. 1. The couchindex (beam arc *j*, with index coordinate at $z_{j}$), being synonymous to *z* coordinate, is used to describe the couch movement of the patient relative to the isocenter.
*D*	Dose in Gray (Gy).
*d*	Depth in cm.
DVH	Dose volume histogram.
*Gx*	Gantry rotational axis.
IMRT	Intensity modulated radiation therapy.
IMST	Intensity modulated sequential tomotherapy, a special category of IMRT.
IVSC	Inverse square correction factor relative to the SAD (source to axial distance).
MIMiC	Multi‐vane intensity modulated collimator. 40 vanes, in two rows, operated in binary mode, are used for radiation intensity controlling.
$〈m_{1/2}〉$	Average field width spanned by the MIMiC at the Gx in the cross‐plane direction.
MU	The monitor unit when beam is on. Normally this MU is calibrated so that 1 MU=1 cGy at the specific depth.
O/P	Output factor, is the linear accelerator (linac) dose ratio at the maximum dose depth, $10\times 10{\text{cm}}^{2}$ field size to any field size.
PCF	Practical Calibration factor.
**r**	A point in 3D space with implicit coordinates (*x,y,z*) (see Fig. 1)
TMR	Tissue maximum ratio, a dose ratio for a given field sizes at any depth to the maximum dose depth.
${\left(t_{i}\right)}_{j}$	Fraction of time that remains opened in the rotation of gantry angle indices i={1,2,3,…,31}⇔{30°,40°,50°,…,θ,…,330°} for a given vane i,j={1,2,3,…,40} of the MIMiC. This is equivalent to the relative beam intensity delivered by vane *j* at gantry angle *i*.
$W_{1\;\text{cm}},W_{2\;\text{cm}}$	The radiation field widths of the MIMiC at 1 cm, 2 cm mode respectively projected along the gantry rotational axis at the isocenter height. These are the widths that are measured at the couch height of isocenter, and the couch should move along the gantry rotational axis for the adjacent arcs irradiation.
θ	Gantry angle.
Θ	Couch angle.
〈Φ〉	The average field size Φ in cm^2^.
%DD	Percentage of depth dose.

**Figure 1:**
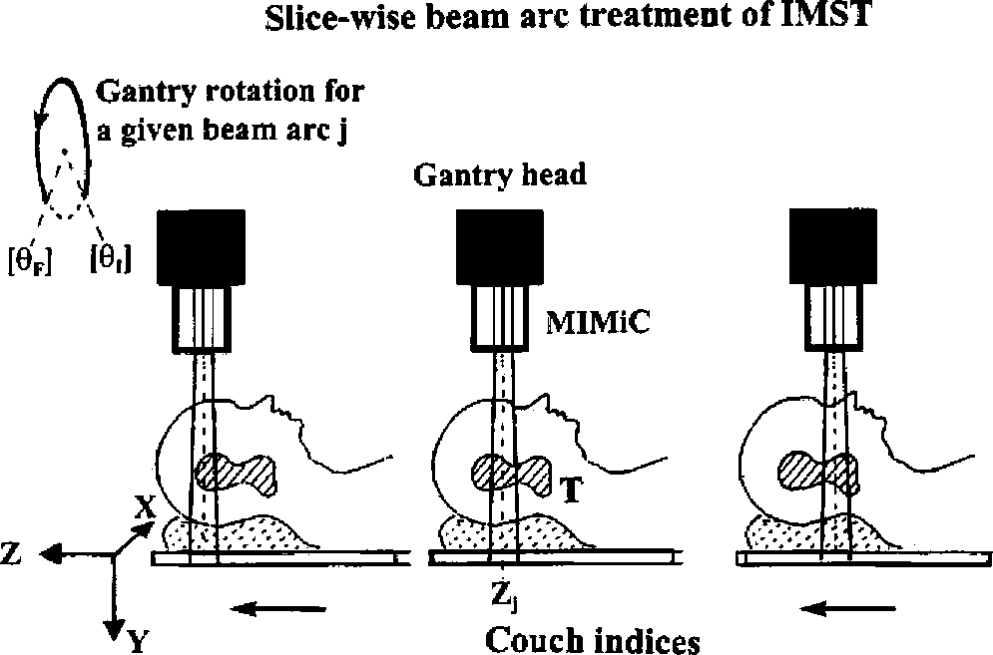
IMST beam ares, couch indices, and the coordinate system, T: target.

### B. Mathematical equations for arc MU calculation

In the gantry rotating IMST, dose delivery to the patient is performed using a multi‐vane switching MIMiC collimator, and is treated arc by arc.^4^ ^,^ ^5^ Each arc has its own MU derived from the inverse plan optimization (Corvus version 3.0, NOMOS Corp., Sewickley, PA). In the present article, we temporarily adopt a hypothesis, to be justified subsequently, that the dose distributed in the transverse plane coplanar to the beam arc central axial plane is overwhelmingly contributed by the “disc” beam arc as implicitly illustrated in Fig. 1. Theoretically, each beam arc contributes some dose to an anatomic location **r**. However, due to the drastical drop‐off of the beam profile beyond a half radiation field width (i.e., either 0.5 $0.5W_{1\;\text{cm}}$ or $0.5W_{2\;\text{cm}}$) along the gantry rotational axis from the beam central line (the location of the couch index coordinate $z_{j}$ of arc *j*), it is reasonable to say that the dose distributed on the transversal plane zj is primarily contributed by the beam arc *j*. The dose contribution from its adjacent arcs is very minor mainly the scattering and leakage. In other words, to evaluate the ${\text{MU}}_{j}$ value for beam arc *j*, we have to identify the *z* coordinate (in the IMST plan) of anatomic CT image so that it matches the beam arc *j* index coordinate $z_{j}$. Thus, at any given anatomic location **r** in the transverse plane $z_{j}$, the dose ${\left(r\right)}_{j}$ and the ${\text{MU}}_{j}$ from this arc *j* have the relationship depicted by the general dosimetry equation.

$$\begin{array}{l} \text{Dose}{\left(r\right)}_{j}=D{\left(r\right)}_{j}={\text{MU}}_{j}\cdot \text{Output}\;\text{factor}{\left(\text{field}\;\text{size}\;\text{ralative}\;\text{to}\;10\times 10{\text{cm}}^{2}\right)}_{j}\\ \;\cdot t{\left(\text{fraction}\;\text{of}\;\text{time}\;\text{vane}\;\text{opened}\right)}_{j}\cdot \text{TMR}{\left(\text{depth},\;\text{field}\;\text{size}\right)}_{j}\cdot \text{Beam}\;\text{profile}{\left(r\right)}_{j}\\ \;\cdot \text{IVSC}+O(\text{scattered}\;\text{dose}\;\text{from}\;\text{adjacent}\;\text{arcs})+O^{\prime }(\text{total}\;\text{leakage}) \end{array}$$

or

(1) $$\begin{array}{l} \Sigma_{i}{\text{MU}}_{j}(\Phi_{i})\cdot O/P{\left(\Phi_{i}\right)}_{j}\cdot {\left(t_{i}\right)}_{j}\cdot \text{TMR}{\left(d_{i},\Phi_{i}\right)}_{j}\cdot \text{Beam}\;\text{profile}{\left(\Phi_{i},r\right)}_{j}\cdot \text{IVSC}\\ \;\;\;\;\;\;\;\;\;\;\;\;\;+O(\text{scattered}\;\text{dose}\;\text{from}\;\text{adj}.\;\text{arcs})+\text{leakage}\\ \;\;\;\;\;\approx {\left\{\text{MU}\cdot (\theta_{F}-\theta_{I}-20{}^{\circ})/(\theta_{F}-\theta_{I})\right\}}_{j}\cdot O/P{\left(〈\Phi 〉\right)}_{j}\cdot {〈t〉}_{j}\cdot \text{TMR}{\left(〈d〉,〈\Phi 〉\right)}_{j}\\ \;\;\;\;\;\;\;\;\;\;\;\;\;\cdot \text{Beam}\;\text{profile}{\left(〈\Phi 〉,r\right)}_{j}\cdot \text{IVSC}+O(\text{dose}\;\text{scattered}\;\text{from}\;\text{adj}.\;\text{arcs})+O^{\prime }(\Sigma_{j^{\prime }}\;{\text{leakage}}_{j}), \end{array}$$

where

i=the running index for gantry angle θ, in 10° step. That is, for example, {1,2,3,…,i,…,31}⇔{30°,40°,50°,…θ,…,330°}

〈Φ〉= The average value of the parameter Φ, etc.

$D{\left(r\right)}_{j}=$ Total dose at the anatomic location **r** corresponding to arc *j* index coordinate $z_{j}$ in the *z* direction.

The factor ${\left\{(\theta_{F}-\theta_{I}-20{}^{\circ})/\theta_{F}-\theta_{I}\right\}}_{j}$ is accounted for the offset of ${\text{MU}}_{j}$ because (Corvus IMST system) the first 10° interval of initial $\theta_{i}$ and final 10° interval of final $\theta_{F}$ of gantry angles, all vanes of MIMiC are still in close status due to the angular acceleration and deceleration of the gantry rotation. $〈\text{TMR}{\left(d_{i},\Phi_{i}\right)}_{j}〉\approx \text{TMR}{\left(〈d〉,〈\Phi 〉\right)}_{j}$, which is valid within the uncertainty ±1% based on our calculation. The zero orders O(dose scattering dose from adjacent beams) and $O^{\prime }(\sum_{j^{\prime }}{\text{leakage}}_{j})$ can be explored by inserting a film at coordinate $z_{j}$ to the humanoid phantom and performing the IMST plan irradiation without the beam arc *j*. For IMST using the NOMOS MIMiC collimator, each term in Eq. (1) cannot be quantified uniquely because of variable field size $\Phi_{i}$, beam intensities $\left(\propto t_{i}\right)$, and depth $d_{i}$. Therefore, the present MU calculation is done implicitly by some “modeled” parameters expressed in the Eq. (1). For simplicity, the subscript *j* for arc *j* will be dropped by default. As mentioned above, the purpose of the present MU calculation is used as a secondary verification of MU derived from the IMST plan for radiotherapy QA. We did not analyze in detail the high order dose components of O(dose scattering) and O'(leakage) for every arc. It is also understood that the dose is accumulated only when the vanes open, which define the dose area.

### C. Estimation of average vane‐open time

**Figure 2:**
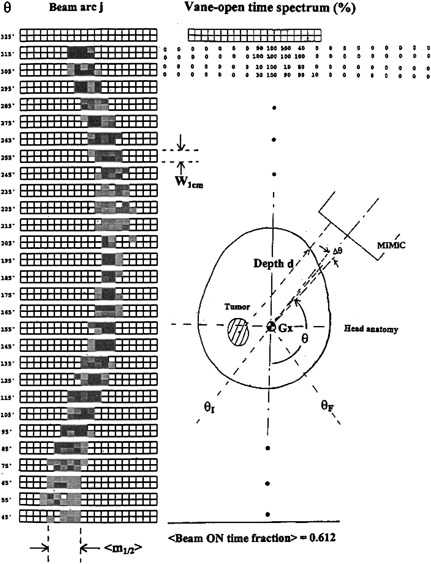
The fraction of each vane's open time. The darker indicates longer open time, while the white vanes indicate all closed. The numerical on the left side shows the numerical fraction of time opened in percentage. The sketch shows the tumor depth variation as gantry rotates during IMST treatment.

The average vane‐open time 〈*t*〉 is quantified using the percentage sum of each ${\text{vane}}_{i}$'s open time fraction $t_{i}$ and averaged over the total vanes number that have involved in the beam intensity modulation and field shaping through the entire arc. This can be illustrated in Fig. 2. The summation is performed from gantry initial angle $\theta_{I}$ to gantry stop angle $\theta_{F}$:

(2) $$〈t〉=\overset{\theta_{F}}{\underset{\theta_{I}}{\Sigma_{\theta }}}\Sigma_{k}t_{k}/\Sigma_{\theta }\Sigma_{k}m_{k}(\theta ),$$

where $t_{i}$'s are numerical percentage printout from the IMST plan and are displayed side by side besides the corresponding vane pattern, and

(3) $$m_{k}(\theta )=\{\begin{array}{ll} 1 & \text{if}\;\;t_{k}\neq 0\\ 0 & \text{if}\;\;t_{k}=0 \end{array}\;\;\;\;\;k=1,2,3,\ldots ,40.$$

This extracted average time 〈*t*〉 is, in fact, the beam on fraction.

### D. Average field size, (A), estimation

For a given specific vane pattern within a 10° interval of gantry rotation, and definition of $m_{k}(\theta )$ in Eq. (3), the average number of vane pairs open across the MIMiC port of the average field size (Φ) during gantry rotation from gantry angle $\theta_{I}$ to $\theta_{F}$, as illustrated in Fig. 2 would be

(4) $$〈m_{1/2}〉=\frac{\Sigma_{\theta }\{{\left[\Sigma_{k=1}{\;}^{40}t_{k}\right]}_{\theta }\cdot [\Sigma_{k=1}{\;}^{40}m_{k}(\theta )]\}}{2(\Sigma_{\theta }\Sigma_{k=1}{\;}^{40}t_{k})}.$$

Thus the averaged field size, for the 2 cm mode MIMiC would be, $〈\Phi =W_{2\;\text{cm}}〉〈m_{1/2}〉{\text{cm}}^{2}$, where $W_{2\;\text{cm}}\approx 3.4\;\text{cm}$ is the index width of 2 cm mode MIMiC along the gantry rotational axis, Gx. The real time fraction, 〈*t*〉, comes from the fact that any observed vane open pattern size is probabilistic in current IMST, and a function of the vane open time. Similarly, for 1 cm mode MIMiC with index width $W_{1\;\text{cm}}\approx 1.7\;\text{cm}$the averaged field size would be $W_{1\;\text{cm}}〈m_{1/2}〉{\text{cm}}^{2}$.

### E. Average depth, 〈d〉

**Figure 3:**
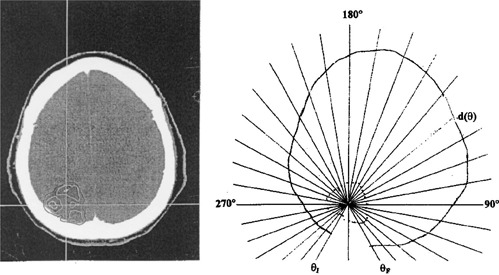
The tumor depth is varied as the gantry rotates. This figure illustrates how the average depth of the tumor is extracted in the present work of MU calculation. The radial lines are drawn diverging from the tumor center. All the radial lines are in equal angular (10°) intervals. Each radial quantifies a depth from the tumor center to the skin. The average depth is obtained by summing up all the radial line depths and divided by the total number of radial line depths.

The average depth 〈*d*〉 for a given anatomic arc is illustrated in the sketch of Fig. 3. An equal angular divergent lines emitting from the common point of the tumor geometric center is fabricated in a transparent sheet. The transparent sheet prints the external anatomic contour and the tumor obtained by overlaying on the transparent sheet on the CT image of some specific couch index coordinate $z_{i}$ along the superior‐inferior (*S/I*) direction. From the transparent sheet, the external anatomic contour is delineated. From the external anatomic contour, the depth, *d*(θ) at a given angle (θ) from the tumor geometric center to the anatomic surface is evaluated by a rule. The average depth, 〈d〉, is thus obtained by the formula,

(5) $$〈d〉=\frac{\Sigma_{\theta }d(\theta )}{\text{Total}\;\text{divergent}\;\text{lines}\;\text{assessed}}\cdot \text{scale}\;\text{factor}\;\text{of}\;\text{the}\;\text{image}.$$

The uncertainty of 〈*d*〉 includes measurement of *d*(θ), noncoincidence of *Gx* and the target center, and adjacent image slices. The overall uncertainty of 〈*d*〉 is expected up to ±5%, taking consideration of contour difference of its adjacent CT image slices.

### F. Average dose, 〈*D*〉, estimation

**Figure 4:**
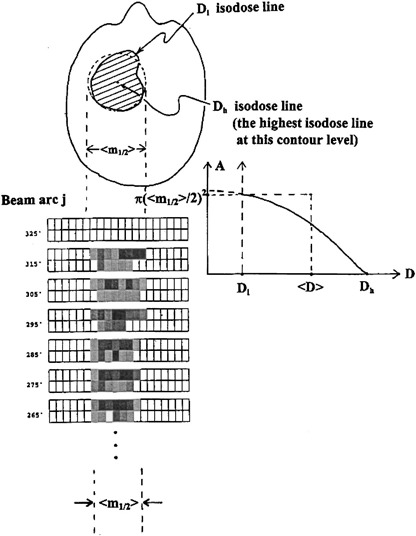
In the present modeled 〈*D*〉, the average dose around the tumor within the field size 〈Φ〉, we assumed $\int D\;da=〈D〉\cdot \pi {\left(〈m_{1/2}〉/2\right)}^{2}$.

To quantify the average tumor dose, $〈D_{j}〉$, on the axial CT image plane directly corresponding to the arc *j*, we use the image contour coplanar to the beam arc *j* at *z* coordinate index $z_{j}$ and display its isodose distribution derived from the IMST plan. The procedure is sketched in Fig. 4. The average dimension of the isodose distribution $〈m_{1/2}〉$ is equal to the average vane pattern size in the transverse MIMiC direction of the arc, as shown in Fig. 2. This isodose distribution is assumed entirely radiated by the shown intensity vane patterns of the arc *j*. Next, in this axial isodose distribution plane of arc *j*, we go to find out the maximum isodose level, $D_{h}$, by assigning an isodose level such that a point or a tiny spot, instead of a spread out isodose distribution appears. Afterwards, we go to find the isodose level $D_{l}$ such that the isodose contour area *A* is equal to $\pi {\left(〈m_{1/2}〉/2\right)}^{2}$. This is based on an assumption that an average width of field size spanned from the MIMiC projected and perpendicular to Gx is $〈m_{1/2}〉$. With this averaged field width $〈m_{1/2}〉$, the arc for a gantry 300° rotation will fabricate an isodose area equivalent to a circle of radius $〈m_{1/2}〉$. Our quantification of the average dose, 〈*D*〉, is the isodose between $D_{h}$ and $D_{l}$ at the arc *j*,

(6) $$〈D〉=\frac{\Sigma_{A^{\prime }}D\cdot \text{pixel}\;\text{area}\;\;A^{\prime }}{\text{Total}\;A}\approx D_{l}+(D_{h}-D_{l})/2=(D_{h}+D_{l})/2.$$

**Figure 5:**
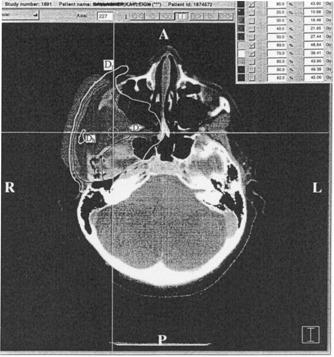
$D_{l}$, 〈*D*〉, and $D_{h}$ isodose distributions.

However, of the 〈*D*〉, a small fraction comes from scattered dose due to adjacent arcs. So, the real dose effectively contributed from the arc *j* beam is 〈D〉−δ〈D〉. This fractional dose δ〈D〉 will be estimated from film measurements. The pursued average dose, 〈*D*〉, is simply the dose of the mean isodose levels of $D_{h}$ and $D_{l}$. This is illustrated in Fig. 5, in which the isodose line of $D_{h}$ is shown as near a spot circle, and isodose line $D_{l}$ is a contour whose area is qualitatively equal to $\pi {\left(〈m_{1/2}〉/2\right)}^{2}$.

### G. Output factor

**Figure 6:**
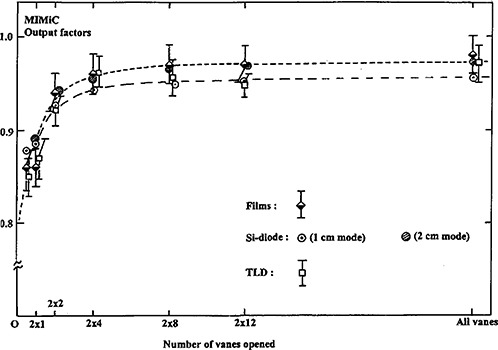
Measured output factors of the MIMiC for IMST beam dose calculation.

The output factors of the IMST beam are shown in Fig. 6 for 1 cm and 2 cm mode of MIMiC respectively. These output factors for specific field sizes are measured using films, Si‐diode, and TLD for mutual consistency. If the equivalent IMST field size is not equal to any measured points shown in Fig. 5, then an interpolated output factor is made by the dashed curve.

### H. Tissue maximum ration (TMR)

The TMR of IMST is deduced from the percent depth dose (%*DD*) curves (not shown in this article), which are stored in the dosimetry base of the Corvus IMST computer. The relationship between TMR and %*DD* is expressed as

(7) $$\text{TMR}(〈d〉,〈\Phi 〉)=\%DD(〈d〉〈\Phi 〉)\cdot (S_{p}^{d}/S_{p}^{m})\cdot {\left[(100+〈d〉)/(100+d_{\max})\right]}^{2},$$

where $\left(S_{p}^{d}/S_{p}^{m}\right)$ is the phantom scattering ratio for the field size projected to different depths of *d* and $d_{\max}$. For simplicity, we have set $(S_{p}^{d}/S_{p}^{m})\approx 1$. The squares of spherical parentheses account for the term IVSC.

### I. Dose contribution from adjacent arcs

**Figure 7:**
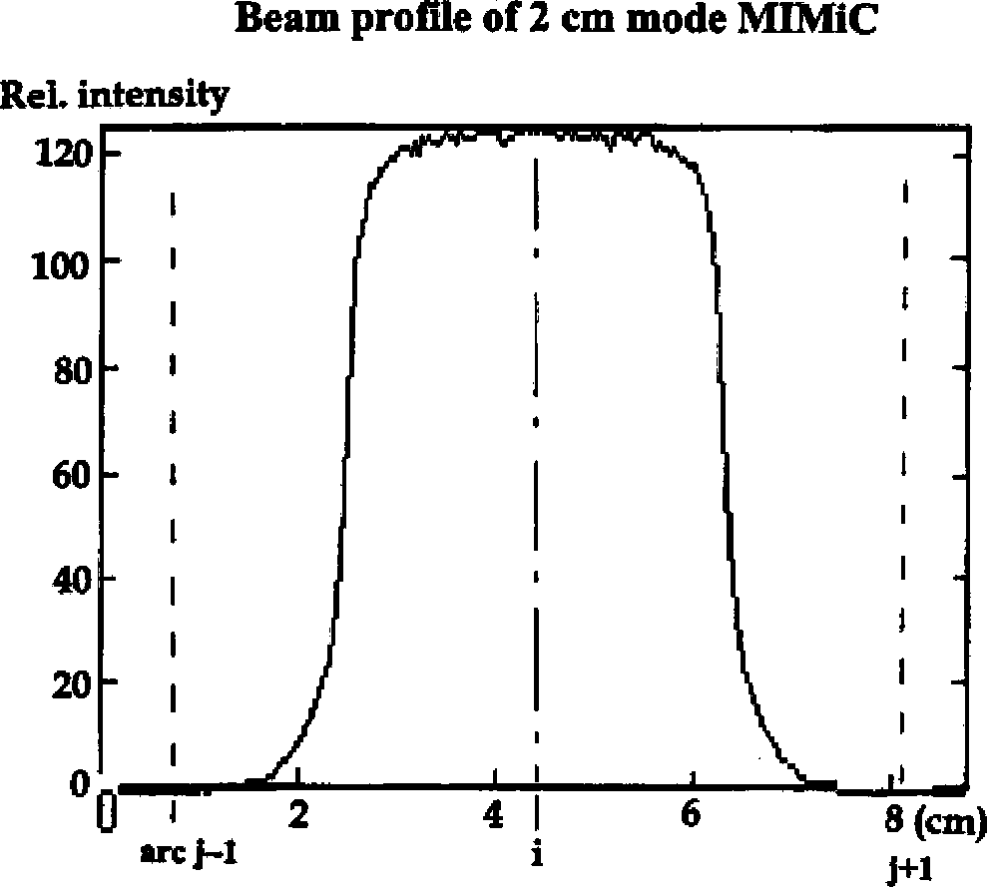
A typical beam profile of 2 cm mode MIMiC. The small penumbra indicates that the radiation extending to the adjacent index positions is negligible.

**Figure 8:**
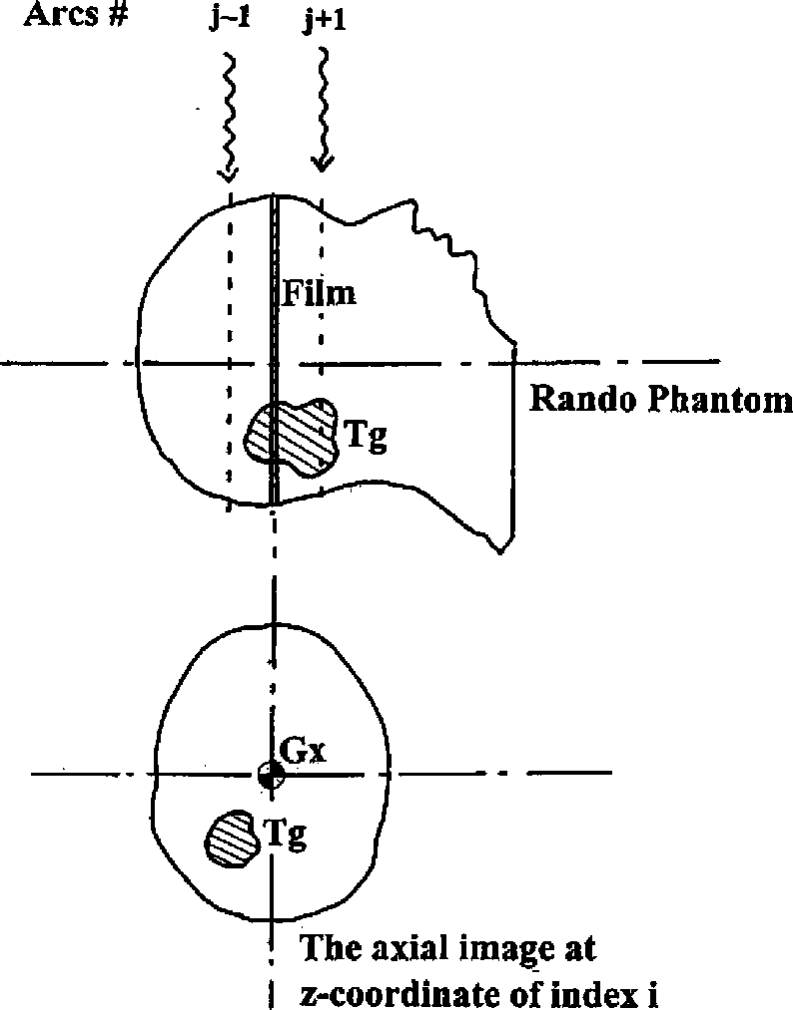
The setup of film and Rando Phantom for the study of the radiation dose contributing to the adjacent axial plane.

In current IMST, the separations between each couch index at coordinates $\left(z_{j}\right)$ and others are in equal distance, either $W_{1\;\text{cm}}$ or $W_{2\;\text{cm}}$ depending on the MIMiC mode used. According to the IMST beam profiles in *z*‐direction, Fig. 7, the radiation intensity at any *z*‐coordinate beyond the beam center by $W_{1\;\text{cm}}$ or $W_{2\;\text{cm}}$ is negligibly small albeit the scattering dose from the adjacent arcs is not totally neglected as expressed in Eq. (1). In order to study the dose contribution from the adjacent arcs to the MU assessing arc, a film (Kodak, X‐Omat V) is inserted to Rando Phantom as shown in Fig. 8. The Rando Phantom is set up in such a manner as described in Tsai *et al*.^1^ The film is located at the beam arc's couch index *z* coordinate. The phantom is then irradiated by IMST plan for arcs j+1,j−1. The dose at tumor target $T_{g}$ contributed from arcs j+1,j−1 is quantified by film scan.

### J. IMST plan dose verification

**Figure 9:**
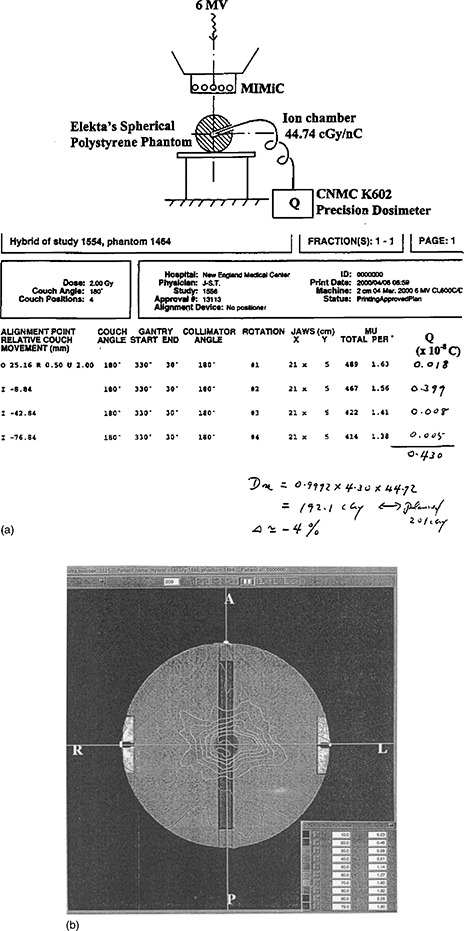
(a) The setup of the spherical phantom and mini‐ionization chamber for absolute dose verification between the IMST plans and that is measured. (b) The dose distribution of the hybrid plan using a Spherical Phantom.

At the moment, we do the IMST arc MU calculations and then compare them with the planned obtained MU. Logically, this is based on the assumption that the MUs derived from the IMST plans are accurate, and correct. To confirm that the MUs from the IMST plans are true, several IMST plans have their MUs verified using a hybrid plan on a spherical phantom. The general IMST hybrid plan technique and its components have been described in Tsai *et al*.^10^ In the present work, a spherical polystyrene phantom used for dose calibration in Leksell Gamma Knife stereotactic radiosurgery facility in conjunction with a Capintec mini ionization chamber (Model PR‐05P, 0.07 cc) are employed [see sketch Fig. 9(a)]. The spherical phantom and mini‐ionization

chamber are used to verify the absolute dose achieved from the hybrid plan [Fig. 9b]. The measured dose from this hybrid plan is compared with the dose expected from the IMST hybrid plan. This will test the reliability and accuracy of the association of the plan MUs with the absolute dose.

## III. RESULTS AND DISCUSSION

### A. Scattered dose from adjacent arcs

**Figure 10:**
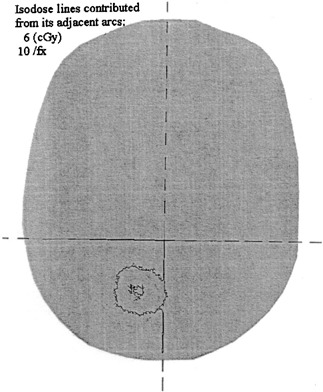
The film dose distribution accumulated from its adjacent arcs.

This has wide uncertainty depending on the number of arcs for each plan, tumor size, and the index width mode of MIMiC. Figure 10 shows one of our studies of the films embedded within the Rando phantom, the scattered dose focused to the area 〈Φ〉, where the δ〈*D*〉 is estimated between 6∼8 cGy/Fx, accounting for δ〈D〉=6∼8/270 cGy or 2∼3% of the 〈*D*〉. In fact, the scattered dose from the adjacent arcs is slightly spread widely over the anatomic space. This implies the quantification of the scattered dose from adjacent arcs is slightly not well defined, and thus incurring a wider range in discrepancy up to 2–4% depending on the target size, target number and dose prescription option to targets. Nevertheless, this discrepancy due to dose scattering from adjacent arcs is smaller than the propagated or accumulated uncertainties from variable fields size $\Phi_{i}$, depth $d_{i}$, time open fraction $t_{i}$, quantified dose $D{\left(r\right)}_{j}$, and O/P due to vane switching.^5^

### B. Calculated MU relative to the planned

**Figure 11:**
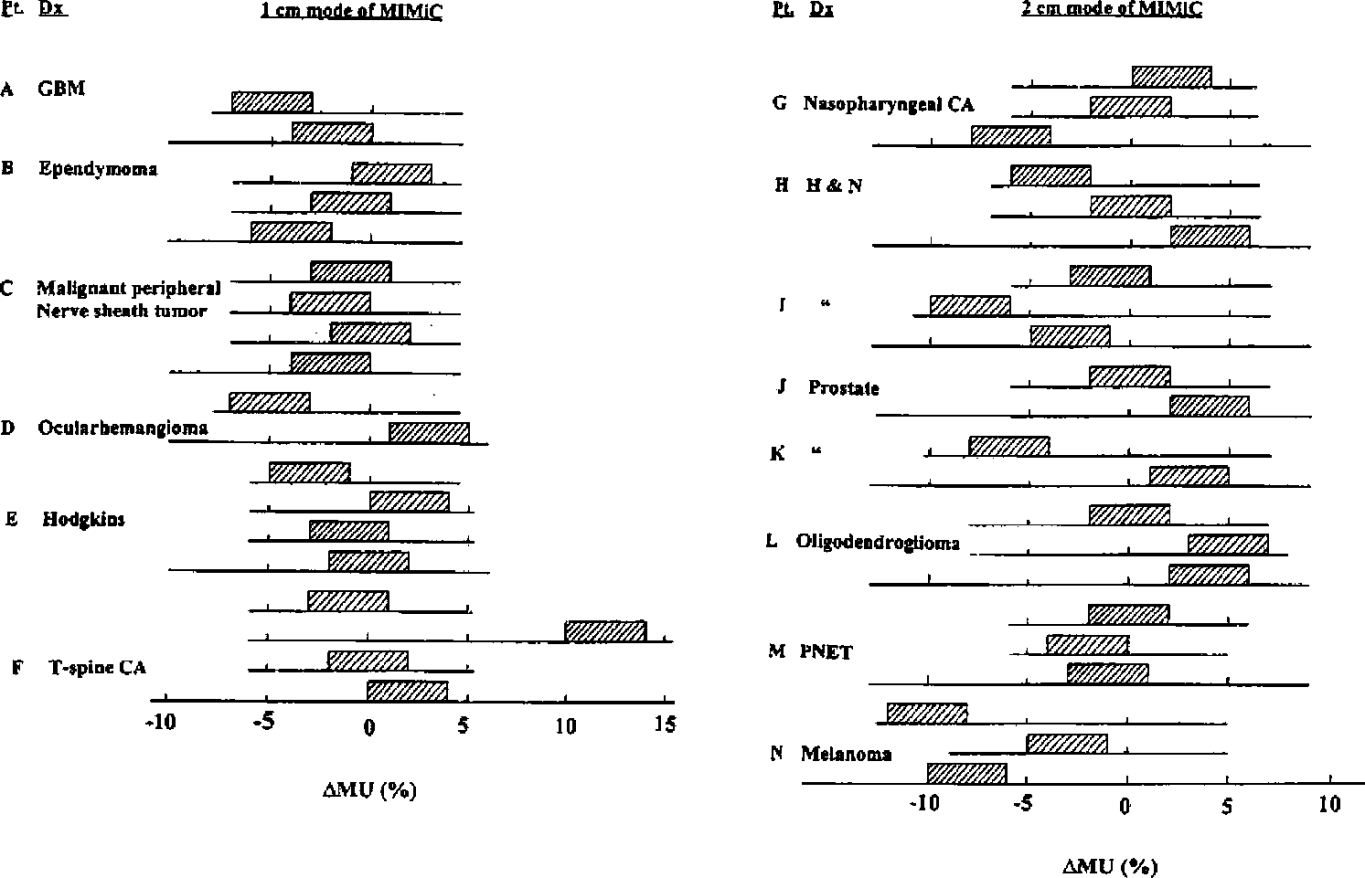
Discrepancies of all the IMST plans.

**Figure 12:**
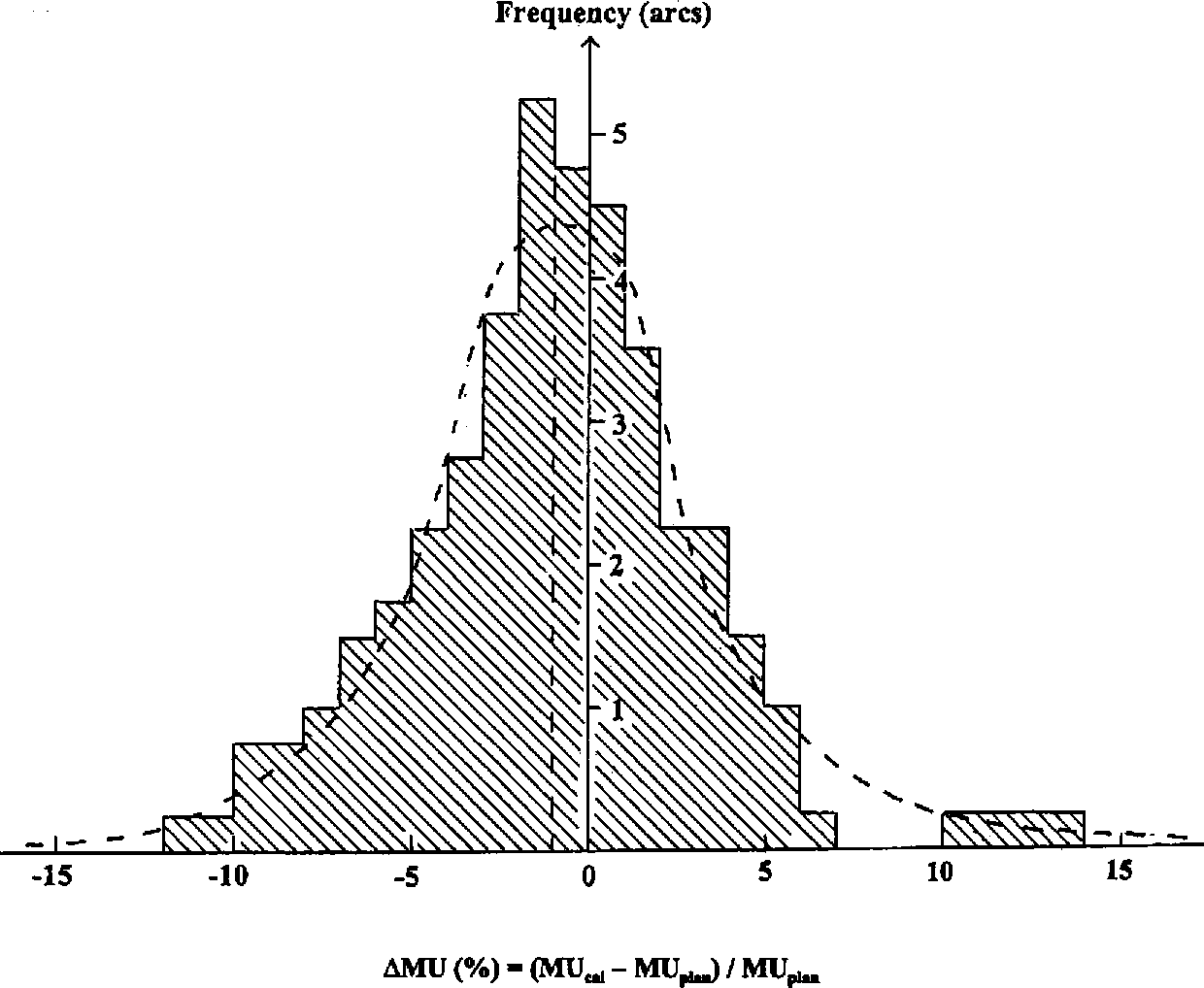
The summed extrapatient MU discrepancies between the calculated MU and that from the IMST planned. The dashed curve is the Gaussian‐fitted curve and the vertical dashed line is the center of the Gaussian distribution.

Fourteen plans were used to test the current “model” MU calculations. All the cases and their MU discrepancies between the present calculation work and the IMST plans were shown in Fig. 11.A typical example of the present MU calculation is shown in the Appendix. In each case, we graphically display its value in percentage +/−2% as experimental error. For example, in the first arc of the GBM patient, the obtained discrepancy –4% will be displayed in the horizontal axis of histogram between –2 to –6%. All such discrepancies are summed up to a combined histogram for the extrapatient MU discrepancy, as shown in Fig. 12. Overall, the average of the total extrapatient MU discrepancy yields −1.0±3.5%, where 3.5% is the standard deviation.

**Figure 13:**
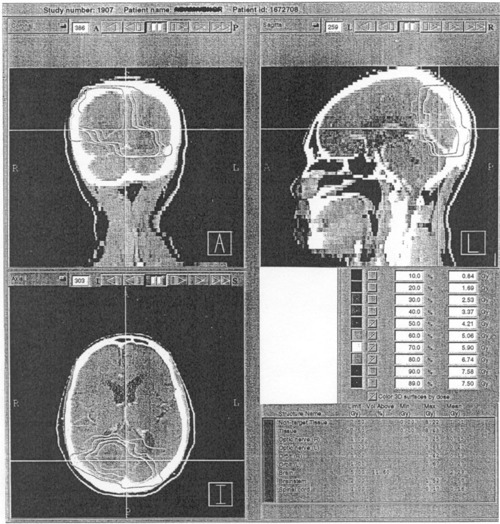
The average dose 〈*D*〉 quantification is sometimes complicated by 2) the high dose gradient of the image which is near the tumor edge, and 2) multiple targets contributed the dose to their high dose areas.

Overall, the global MU discrepancy between the present calculations and the planned is in agreement. The wide spread of discrepancy is attributed to the following reasons:

1) Average dose, 〈*D*〉, uncertainty. This 〈*D*〉 is quantified based on the “model” that the dose volume histogram (DVH) between the region of $D_{l}$ and $D_{h}$, shown in Fig. 4, is triangle in shape. As a result, the 〈*D*〉 is a mean value of $D_{l}$, and $D_{h}$. Any DVH deviated from the aforementioned shape will alter the 〈*D*〉, and automatically change the calculated MU value. In several cases, the beam index position lies either near the tumor edge, where the dose distribution shows a high gradient, or the between two tumor targets (see Fig. 13), which further complicates 〈*D*〉 quantification. In Fig. 13, the arcs delivering dose for upper target “share” portioned MUs from the arcs that deliver dose to lower target, and verse versa. Under such circumstances, higher discrepancy between our calculated MUs and the IMST planned is expected because of less accurate dose quantification from the arcs being responsible for the dose coverage of the lower target.

2) Average fields size dimension, 〈Φ〉, uncertainty. The 〈Φ〉, being probabilistic in nature as mentioned above and depicted in Fig. 2, has incorporated some extent of uncertainty. The uncertainty propagates subsequent uncertainty of the average dose assessment.

3) Index *z* coordinate uncertainty for 〈*D*〉 quantification. In all 41 extrapatient IMST arcs calculations of MUs, we evaluate the 〈*D*〉 from the isodose distribution of the plan on an axial image at the *z*‐coordinate specified by the IMST treatment sheet. This is based on the assumption that the vane pattern opened in MIMiC, no matter 1 or 2 cm mode, includes both rows. This however, is not true in some cases near the tumor edge, where only one row, either superior or inferior of the MIMiC is involved for beam field intensity modulating and shaping. In order to minimize the *z*‐coordinate deviation for one row MIMiC open, the axial image of its *z* coordinate is adjusted. For example, in 1 cm mode MIMiC, if the arc index *z* coordinate is $z_{1}$, and only the superior row is involved, then the 〈*D*〉 quantification is not at axial image of $z_{1}$, instead, it is of $z_{1}+0.42\;\text{mm}$.

4) The average dose, $〈D_{j}〉$, in the CT image of $z_{j}$, being overwhelmingly contributed by the beam arc *j*. This is true for single couch angle setup. However, for multiple couch angle setup, the same $〈D_{j}〉$ may be crossed over by beam arc *j′* of couch angle Θ′, and beam arc *j″* of couch angle Θ″ setup. The portion contributions to $〈D_{j^{\prime }j^{\prime \prime }}〉$ from beam arc *j′* and *j″* is more complicated than we expected. Therefore, the present quasi‐calculation of MU using Eq. (1) is infeasible for multiple couch IMST setup.

5) As aforementioned, we have simplified the present calculation in Eq. (7) that the phantom scattering ratio $S_{p}^{d}/S_{p}^{m}\approx 1$. This may incur an uncertainty up to about ±2%.

6) In our MUs calculations using Eq. (1), no heterogeneous density correction is made. Nevertheless, NOMOS's Corvus planning software, albeit its potential, still remains unproven for the correction. Thus, the MU discrepancy due to density heterogeneity remains inconclusive. Detailed dose calculation algorithm of Corvus was reported by Bleier *et al*.^18^

For a given patient, and given beam arc (i.e., given couch index), MU discrepancy between the present calculations and the planned does raise comparative uncertainty. It doesn't mean any flaw of the present methodology. Instead, it mainly attributes to the modeled and probabilistic quantities for some parameters proceeded in the present work.

### C. Dose verification of hybrid plan

The result of absolute dose measurements from spherical polystyrene phantom of eight hybrid plans is listed in Table I. The averaged discrepancy between measured doses and the planned is less than –1%, indicating, overall, in good agreement. The standard deviation, roughly about 4%, displays a slight spread of the measured data relative to the planned ones. This is due to the fact that some of the locations of dose measurements within the phantom are in high dose gradient region that makes the discrepancies between the measured ones and the planned expectations slightly high. Any discrepancy if exists, however, may include many factors. First, in many cases, the hybrid plan isodose distribution near the spherical phantom center, where the ionization chamber is located, is in a high dose gradient. Second, IMST planning software uses a Practical Calibration Factor (PCF) to scale the absolute dose readings. PCF was assigned by actual dose measurements within the humanoid phantom for several simulated targets and compare with the planned ones. PCF is slightly correlated to the target's location relative to the gantry rotational axis (Gx). In our initial assignment of PCF during the commission, several targets at various locations were measured to offset the deviation and obtain an average PCF. However, the patient's tumor locations are still randomly distributed within the anatomic space, and it is not surprising that there exists some dose uncertainty between the real measured dose and the planned one due to slight deviation of PCF. Third, the ionization chamber, more or less, has stem effect for dose detection efficiency on the IMST beams.

**Table I:** Absolute dose verified from the hybrid plans of the spherical polystyrene phantom.

Trial #	Verified dose (Gy)	Hybrid planned dose (Gy)	Ratio (verified/hybrid)
1	1.36	1.45	1.06
2	3.23	3.21	1.01
3	1.92	2.01	0.96
4	5.09	5.34	0.95
5	5.00	5.04	0.99
6	1.97	1.91	1.02
7	1.64	1.74	0.97
8	2.42	2.44	0.992

## IV. CONCLUSIONS

As IMST dose, dose distributions, and MU have been extensively verified with measurements in anthropomorphic and other phantoms using the Corvus hybrid plan tool, the quasi‐independent monitor unit verification may be applied to simplify QA cost overhead in the implementation of IMST. The current MU calculation can be used for an IMST treatment second check and thus as a tool of QA for IMST dose delivery. Because the MU calculation cannot vigilantly pick up other hidden errors, such as patient setup during treatment, hybrid phantom verification will continue to be performed as supplementary for patients not belonging to classes of anatomy and/or delivery techniques already verified in phantom for prior patients.

## ACKNOWLEDGMENTS

We greatly acknowledge the NOMOS Corporation for providing information that leads to get MIMiC time open fraction. Additionally, we thank for Mark J. Rivard, Ph.D. for English corrections, and Ali Mahmoudieh, M.D. for some calculations checking.

## Figures and Tables

**Figure 14:**
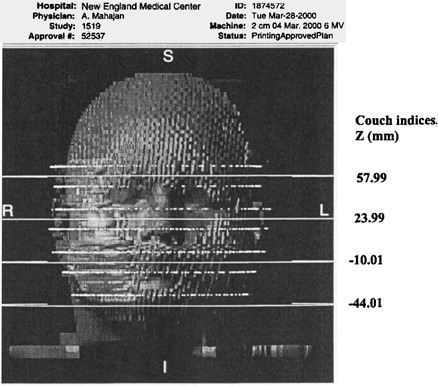
IMST beam arcs and its couch indices.

**Figure 15:**
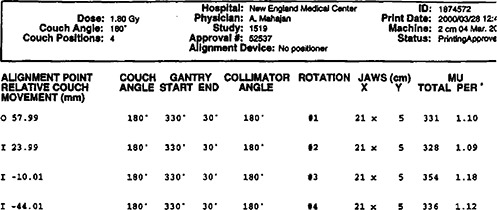
IMRT treatment sheet.

**Figure 16:**
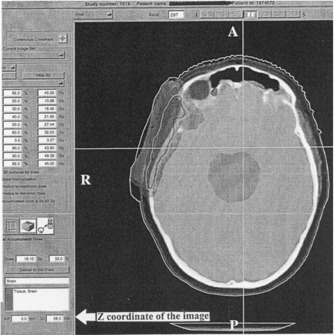
The CT image, including its isodose distribution, closest to the couch index location (Z=57.99 mm) of the beam arc #1.

**Figure 17:**
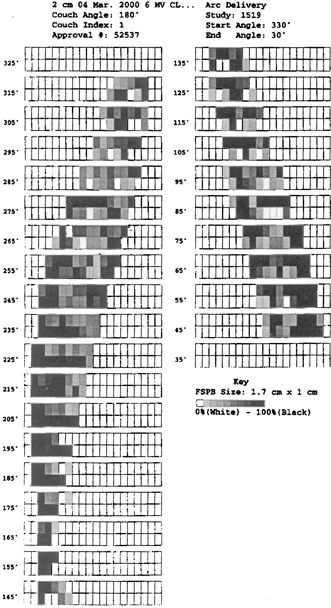
Vane patterns of beam arc #1 at different gantry angle.

## ONE EXAMPLE OF QUASI MU CALCULATION FOR BEAM ARC #1 OF IMST PLAN FOR PT. G.K.

Step 1. Identifying the beam arcs and their corresponding couch indices in the *Z* direction, as shown in Fig. 14.

Step 2. Print a copy of the treatment sheet of the IMST plan #1519, as shown in Fig. 15. The treatment sheet displays the “alignment point relative couch movement” of each beam arc (couch) indices in terms of the *Z* coordinate. The couch index of beam arc 1 is Z=57.99 mm. This means that the transverse central beam of the MIMiC is in coincidence with transverse plane Z=57.99 mm, i.e., the CT image of Z=57.99 mm, for gantry 30° to 330° rotation. The MU for this beam arc is 331.

Step 3. The CT image whose *Z* coordinate closes most to the couch index of (beam arc #1) 57.99 mm is the axial CT image #297 (the image registration number from Corvus' planning algorithm) at Z=58.0 mm, see Fig. 16.

Step 4. Calculation of the average depth 〈*d*〉 of the target based on the CT image Fig. 16 is 6.06 cm. The calculation steps are illustrated in Fig. 3 using Eq. (5).

Step 5. Vane patterns of beam arc #1 is shown in Fig. 17, from which the average fraction time of vanes open is $〈t_{1}〉\approx 0.727$, shown in Fig. 2 from Eq. (2).

Step 6. From the vane pattern of Fig. 17, we are able to calculate the 〈m〉=6.0 cm using Eq. (4) as shown in Fig. 4.

Step 7. The average dose 〈*D*〉 isodose line whose area $\approx \pi {\left(6.0\right)}^{2}{\text{cm}}^{2}$ is 180.2 cGy/fx, shown in Fig. 16. From this dose, we assume 4∼5% is attributed from the scattering doses from beam arcs #2, 3, and 4 and radiation leakage.

Step 8. The O/P factor for field size of $3.4\times 6.0{\text{cm}}^{2}$ would be 0.955. The beam width, i.e., the field length projected with MIMiC field port along the gantry rotational axis is 3.4 cm for 2 cm MIMiC set‐up, which was used for the IMST plan #1519.

Step 9. The TMR $(d〈d〉=6.0\;\text{cm},3.4\times 6.0{\text{cm}}^{2})=0.764$.

Step 10. Beam profile central‐axis‐ratio ≈1, i.e., assuming the average of the dose distributed to the adjacent axial CT image #297 by the beam arc #1 has a beam profile central‐axis‐ratio of 1.

Step 11. MU calculated=180.2×0.95/(0.955)×0.764×0.727×0.931)=346.7≈347 MU using Eq. (1).

Step 12. The MU of this beam arc #1, derived from the IMST plan #1519, is 331.

Therefore, the discrepancy is ≈+5%.
